# Nest site selection and fidelity of European pond turtle (*Emys orbicularis*) population of Babat Valley (Gödöllő, Hungary)

**DOI:** 10.1186/s12983-024-00541-3

**Published:** 2024-08-12

**Authors:** István Kiss, Gergő Erdélyi, Borbála Szabó

**Affiliations:** 1 Department of Zoology and Animal Ecology, Hungarian University of Agriculture and Life Sciences, Páter K. Street 1, Gödöllő, 2100 Hungary; 2 General and Theoretical Ecology, UFT, University of Bremen, Leobener Str. 6, 28359 Bremen, Germany

**Keywords:** Habitat features, Inter-nest distances, Migration distances, Nest density, Nesting area, Hatching success

## Abstract

**Background:**

The conservation of aquatic and semiaquatic turtles requires knowledge of the area and vegetation structure of habitat used for nesting, and nesting migration route. We aimed to survey the effects of habitat features to the nest site selection, nesting success, and test the possibility of nest site fidelity. Our study was carried out at 10 different nesting areas, with special emphasis on data from returning females in a pond system in Hungary between 2014 and 2017.

**Results:**

Most nesting attempts were found in closed sand steppes, uncharacteristic dry and semi-dry grasslands habitat patches. The principal component analysis (PCA) showed that increased sandy soil cover, sunlight and slope were important variables in nest site choice. The increasing PCA first axis score significantly increased the chance of an emergence. The degradation of open steppe vegetation, occurrence of weeds, invasive and disturbance tolerant species have a negative effect on the selection of nest sites. We observed that 96.55% of nests were located within 20 m south of a pine forest at preferred nest site at pond 5, which provided the right incubation temperature through partial shading. The returning females nested significantly closer to the northern pine forest than the single clutch females. Most probably the returning females already has the necessary experience to select the right nesting site. The individually marked females did not choose new nesting areas during the monitored years which suggests nesting area fidelity, but we did not find nest site fidelity.

**Conclusion:**

The maintenance of mosaic habitat structure, slowing down the succession process at the nesting area should be basic priorities in European pond turtle conservation programs. We suggested a spatial and temporal scheduling of land management and agricultural work to the local farmers. If the actual nest site is in an agricultural area, all work should be avoided throughout the year. Agricultural machinery should avoid the migration routes of adult turtles and emerged hatchlings during the concerned period. Under strong predation pressure, predator control should be carried out, and use nest protection.

**Supplementary Information:**

The online version contains supplementary material available at 10.1186/s12983-024-00541-3.

## Background

Nest site fidelity is well known among birds (e.g. [25, 63]), and even reptiles, mainly in sea turtles (e.g. [41, 76, 78]), but also in freshwater turtles [21, 22, 33, 46, 51, 61].

Freshwater turtles need to find an adequate egg laying site near the aquatic habitat to maintain a healthy population with successful breeding. Optimal habitat conditions for an egg laying site are already described for many species, such as European pond turtle (*Emys orbicularis*) [20, 79]. The aims of the optimal nest site selection are to minimize the mortality of females and maximizing the breeding success [10, 36, 68, 72]. The habitat features of the egg laying sites and nest’s microenvironments including the soil conditions, thermal conditions and area of the canopy cover affect the incubation temperature, the duration of embryonic development, the timing of hatching, the hatchlings’ sex ratio, the offspring fitness [11, 30, 36, 38, 71, 78].

The nest site fidelity concept can be interpreted over a very wide spatial scale, from the exactly same site in space to several kilometres of coastline. Repeatedly returning to the same nesting area and nest site under unchanged environmental conditions can have a number of benefits, such as reducing the energy required by females to search new nest sites, reducing potential risks of mortality of gravid females and increasing the safe hatching of offspring [8]. Nest site fidelity can be defined best as the proximity of repeated nesting of a given female to its previous nests. However, depending on the size of the potential nesting area, the distance is difficult to determine and may vary between and within species [10, 71].

Non-random nesting and nest site fidelity were documented in freshwater turtles, for example in painted turtles (*Chrysemys picta)* by Lindeman [33] and Rowe et al. [61], in Blanding’s turtles (*Emydoidea blandingi*) by Congdon·et al. [12], and western pond turtle (*Emys marmorata*) by St. John [71]. However, it is questionable whether nest site fidelity is primarily due to good microhabitat selection, or whether returning where they emerged as hatchlings may a play a role too [45]. Although, spatial data on repeated nesting in the European pond turtle are scarce, and the published data differ in methodology.

Once out of the water, the length of the migration route can vary greatly depending on habitat conditions. Female turtles often find a suitable place to lay eggs within a few hundred metres [72], but sometimes they may have to migrate for kilometres [9, 64–66]. Migrating long distances enlarges the predation pressure, and if the migration route is crossed by roads, the risk of mortality increases significantly [67, 73]. In such cases, conservation management may be necessary, or even the creation of new nest sites with suitable microhabitats closer to the aquatic habitats [56].

Conservation measures should take into account that the habitat parameters of nesting areas used by long-lived turtles may change over time, so females may need to find new nest sites [47]. European pond turtle experiencing demographic declines throughout its geographic distribution due to a variety of factors, such as habitat fragmentation and loss, degradation of wetlands, and nest depredation [24]. Semiaquatic turtles, as European pond turtle, with biphasic lifestyle complicate the management of protected areas, because they require both good quality freshwater habitat for foraging and basking and terrestrial habitat with suitable routes of migration and open, sunny areas and low plant cover for successful nesting [15, 60, 74, 79]. One of the driving parameters for turtle dispersion activity could be the salinity of the water [1, 35]. Ficetola et al. [20] state that woodlands strongly influence adjacent wetland features and may be important for turtles’ terrestrial activities, such as dispersal and nesting. Recognizing the specific nature of the species, conservation measures have been developed in many European countries that focused mainly on nest protection, re-introduction, and habitat maintenance or restoration [7, 18, 26, 40, 62].

In some areas of Hungary, the number of European pond turtles decreased, however, it is difficult to estimate the extent of the decrease in the absence of detailed studies at national and local level [18]. Most studies in Hungary have dealt with empirical reviews of morphological characteristics, life cycle, population structure and dynamics and genetic diversity [2, 17, 37, 50]. Recent studies have examined basking site preference, basking activity, nesting activity, reproduction success and the predator effect [14, 15, 29, 57].

We aimed to answer to the following questions: (1) Did the habitat features influence the number of successful nesting attempts and hatchling emergence within 100 m radius of nest sites? (2) Did the density of nesting attempts and the inter-nest distances differ at the nesting areas? (3) What were the nesting migration distances from the ponds to the nest sites? (4) How was the selection of nest sites influenced by pine forests bordering the nesting area? (5) Could be observed nesting area and nest site fidelity for returning females?

## Materials and methods

### Study area

**Fig. 1:**
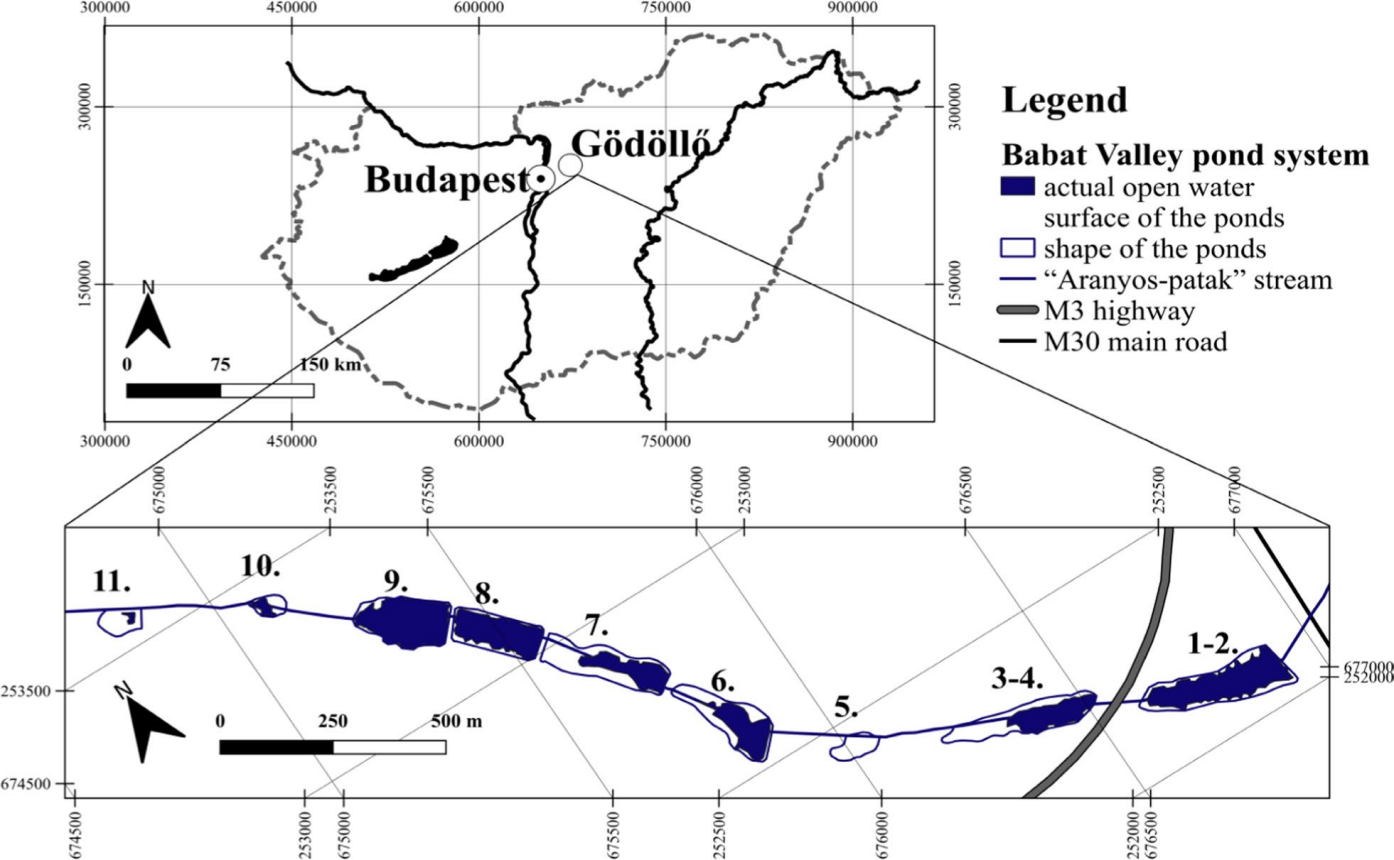
Position of the ponds in Babat Valley (near Gödöllő, Hungary) in a large-scale map. The ponds are indicated by their numbers. The map sections of Hungary at a scale of 1:3,800,000, the pond system 1:10,500. Colours are given to the online version of the manuscript

**Fig. 2:**
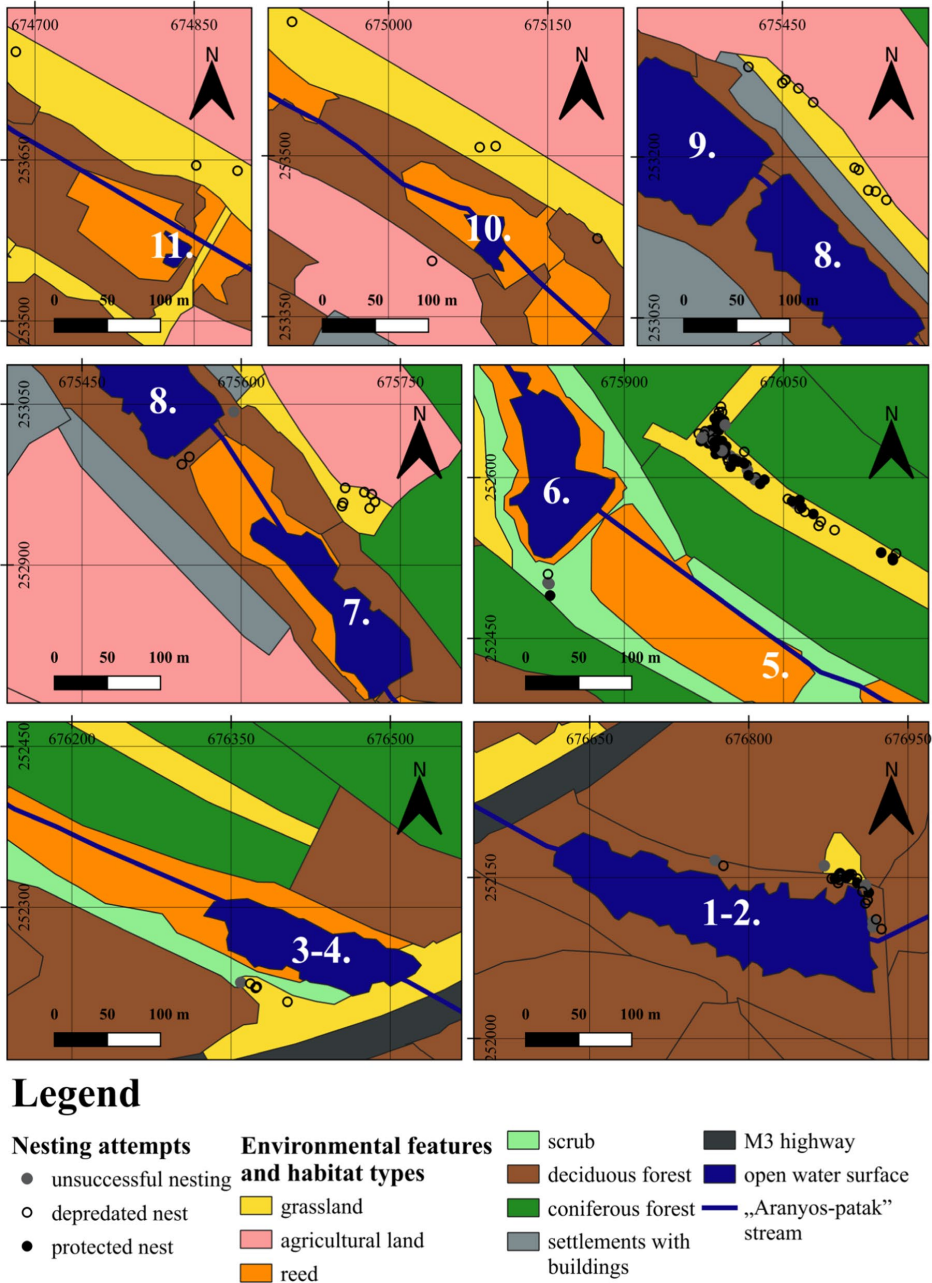
Environmental features and habitat types around the nesting areas of the European pond turtle in the Babat Valley. The ponds are indicated by their numbers. The map sections are at a scale of 1:4200

Our survey was carried out in the area of Babat Valley pond-system (Fig. 1) that is located on the outskirts of Gödöllő (47°36′N; 19°22′E), northeast of Budapest, Hungary. The total open water surface of the pond system is about 7.6 ha. The ponds are surrounded by the mosaic of deciduous and conifer woods, grasslands and agricultural fields (Fig. 2). On the north side of the ponds, there are mainly agricultural fields, pine forests, sand steppe grassland associations, and uncultivated lands between the fields. Whereas on the south, there are mostly deciduous forests. An asphalt access road runs on the south side of the ponds. Ponds 1‒2, 8 and 9 are currently under non-intensive and periodic fishing. In the vicinity of ponds 7, 8 and 9, there is a former goose breeding farm, as well as buildings of warehouses and fishing guest houses. The surfaces of the ponds were covered by reeds to a varying degree. The open water surface of ponds 3‒4, 6 and 7 was reduced to the eastern half of the pond. In pond 10 and 11, open water surface was very restricted, while the entire surface of pond 5 was covered by closed reeds.

We have defined the term “nesting area” as the general area of habitat used for nesting. “Nest site” defined as an exact location of the nest within the nesting area, identified by geo-coordinates. The term “nesting attempt” means that the female turtle has started nesting, which may be unsuccessful when no eggs are laid, or successful when the turtle has laid her eggs and covered the nest. Depredated nest means, that predator dug up and robbed the eggs, while protected nest means, that nest was protected with a metal square grid. We used aboveground metal square grids 30 × 30 cm in size with a mesh size of 3 cm for nest protection [62]. “Returning female” means that a female was observed nesting twice in the same year (“double clutching”) or in one or more consecutive years. Nesting twice in a year or nesting again in consecutive years always mean successful nesting attempts. In our earlier study we observed that the number of days elapsed between the first and the repeated egg-laying in the same year was similar in the 4 year, the mean length was 22.5 ± 1.3 days (range, 21‒24 days) [29]. A “single clutch” means that the nesting of a female was observed only once and not again, or observed nesting without identification of the female.

### Data collection

We registered the nesting attempts of female turtles at their nesting area in the spring of the years 2014–2017 (Additional file 1: Table S1). Earlier we identified two “preferred nesting areas” where the number of nesting attempts were the highest around the pond system [29]. The largest one on the band of steppe grassland associations at pond 5 (“most preferred nesting area”), and a small patch of grassland on the shore of pond 1‒2. We checked these preferred nesting areas almost daily (2014: 20, 2015: 39, 2016: 24 and 2017: 20 times), starting at dusk (Additional file 1: Table S2). We patrolled the whole areas of the two preferred nesting areas in every half hour during the night surveys in the nesting season to maximise the number of caught and marked females. Once females were finished laying eggs and had covered the nest, we caught and marked them on the marginal scutes of the carapace using a rasp, and protected their nests immediately (see details in [29]). We marked protected nests with a serial number on a plastic ticket. In the case of other nesting areas, we searched for nesting attempts (2015‒2017) during the day every week or two and could detect only depredated nests due to lack of nest protection (Additional file 1: Table S2). We also marked all unsuccessful and depredated nests everywhere to prevent their re-assessment. We have saved the EOV coordinates of all unsuccessful, depredated and protected nests with Meridian Golds, later Garmin eTrex 30 devices.

We inspected the protected nests weekly during the summer. A few weeks before expected emergence in 2015‒2017, we attached a metal cage (5 mm mesh) above the metal grid, which prevented the emerged hatchlings from leaving immediately and provided them protection from predators. After placing the protective cages, we checked for emergence daily, usually in the late morning. After emergence, we removed the cage and grid, excavated the nest, and counted unfertilized eggs, eggs with dead embryos, and any dead hatchlings which remained in the nest cavity. “Emergence success” means = number of emerged hatchlings/number of all hatched neonates [29] (Additional file 1: Table S1).

We estimated the degree of sunlight at the nesting areas based on the percentage of the site covered by individual trees or shrubs that cast shade at varying locations depending on the size of the canopy, thus reducing sunlight. In the majority of cases, trees and shrubs were scattered on or adjacent to nest sites, so sunlight levels were generally 80‒90%. However, three nesting areas (at ponds 5, 10 and 11) were free of trees and had 100% sunlight.

### Mapping

We used QGIS 3.16.3 [58] to create maps, calculate vegetation type cover and distances. We displayed the nest based on the EOV coordinates. The distances between the nests measured with standard distance matrix by nesting area per year. We used the NNJoin 3.1.3 module to determine the distance of nests between the nearest shores of ponds and the distances between nests and black pine plantations in the nesting area of pond 5. The size of the actual nesting area was interpreted as the area occupied by the edge of the nest sites observed in a given year or in total over the four years.

To determine the percentage of habitat types within 100 m radius of the nest sites we used a local habitat map [75], based on the National Habitat Classification System (ÁNÉR categories) [4]. The Fig. 2 shows the main habitat categories surrounding the nest sites. Within these, further subcategories could be distinguish and have been used in the analyses: grassland (uncharacteristic dry grassland, closed sand steppes with uncharacteristic dry grassland), deciduous forest (sessile oak-hornbeam forests, scattered native trees or narrow tree lines, uncharacteristic pioneer softwood forests and plantations, uncharacteristic hardwood forests and plantations, not-native deciduous forests and plantations mixed with native trees, clear cut areas, *Robinia pseudoacatia* plantations, scattered trees or narrow tree lines of non-native trees), coniferous forest (*Robinia pseudoacatia* plantations + scotts and black pine plantations, scotts and black pine plantations), reed (eu- and mesotrophic reed and *Typha* beds, non-tussock tall-sedge beds), agricultural habitats (annual intensive arable fields, new abandonments of arable lands).

### Statistics

We used R 4.0.3. [59] to all statistical analysis. To investigate the habitat features of the different nesting areas we carried out PCA (Principal Component Analysis) with the percentage of different habitat types in the hundred-meter radius around each successful nesting sites, the percentage of the slope, and the percentage of sunlight. We used every variable in percentage format to standardize them for the analyses. If a variable was homogeneous in one dataset, or had zero weight on the first axis, we omitted it from the analysis. We analysed data in three layers: successful nesting attempts (protected and depredated nests), protected nests, nests with successfully hatchling emergence (1–100% hatchling emergence success). We did not include unsuccessful nesting attempts because their causes could not be related to the habitat features included in the analysis. Also, we present all three cases in Table 1 with the first axis and the corresponding Eigenvalues.

**Table 1:** Effect of habitat types of 100 m radius and environmental features on the successful nestings (protected and depredated nests), protected nests, and nests with successful emergence

	Protected and depredated nests		Protected nests		Nests with successful emergence	
Habitat types and environmental features	Axis 1	Eigenvalue	Axis 1	Eigenvalue	Axis 1	Eigenvalue
Closed sand steppes + Uncharacteristic dry and semi-dry grasslands	0.363	6.644	0.316	9.228	0.313	9.188
Uncharacteristic dry and semi-dry grasslands	− 0.272	2.186	− 0.324	1.000	− 0.324	0.513
Wet and mesic pioneer shrub	0.178	1.269	0.128	0.598		
Scattered native trees or narrow tree lines	− 0.275	0.765	− 0.324	0.135	− 0.319	0.243
Black locust plantations	− 0.291	0.472	− 0.318	7.730 × 10^– 04^	− 0.318	7.730 × 10^– 04^
Black locust plantations + Scots and black pine plantations	0.138	0.267	0.326	4.983 × 10^– 04^	0.326	4.983 × 10^– 04^
Scots and black pine plantations	0.364	0.203				
Scattered trees or narrow tree lines of non-native tree species	− 0.139	0.096				
Uncharacteristic or pioneer softwood forests			− 0.227	2.505 × 10^– 02^	− 0.273	3.864 × 10^– 02^
Non-native deciduous forests and plantations mixed with native tree species			− 0.320	1.249 × 10^– 02^	− 0.320	1.249 × 10^– 02^
Annual intensive arable fields	− 0.137	0.046				
Standing waters	− 0.374	0.030	− 0.327	4.697 × 10^– 05^	− 0.328	2.280 × 10^– 06^
Sunlight	0.367	0.021	0.321	2.646 × 10^– 08^	0.319	4.717 × 10^– 17^
Slope	0.372	0.002	0.321	6.617 × 10^– 24^	0.319	0

The Table does not include habitat categories that were not included in the PCA analysis due to lack of impact

We checked the assumption of the linear models with the model assumptions plots (residual normality and variance homogeneity: QQ plot, residual plots), and the outliers were checked with a Cook-plot. Multicollinearity was checked with Pearson’s correlation table and was accounted for with either the usage of the PCA axis or adding the variables separately to the models. The Pearson’s correlation matrices of the simultaneously occurring continuous variables are in Additional file 1: Tables S3, S4, S5 and S6.

To investigate the habitat features influence on the number of successful nesting attempts and hatchling emergence, we performed the following analyses. Because hatchling emergence data were zero inflated, we analysed the success of emergence (there were emergence vs. there was no emergence) separately from the extent of the success (if there was emergence, how large it was). We analysed the effect of the first axis of PCA on the emergence success with a *glm* and *glmer* (lme4 package; [3]) with binomial distribution. We used the nesting area as a random factor (aka. random subject) in the *glmer*. We used both mixed effect and fixed effect model to test whether there is an effect of the sites or not. We tested the effect of the first axis on the hatchling emergence success with *lm* and a *glmer* with normal distribution to see the effect of the nesting area as random factor.

We analysed the general environmental factors effect on the number of nesting attempts with *lm*, so we had the following models: number of nesting attempts ~ slope, number of nesting attempts ~ sunlight; number of nesting attempts ~ exposure. We analysed the effect of the size of the nesting areas on the density of nesting attempts with *lm*.

To analyse the relationship between the inter-nest distances considering the different size of the nesting areas (Additional file 1: Table S7), we used the corrected inter-nest distance. We calculated the corrected inter-nest distance by dividing the inter-nest distance with the longest inter-nest distance measured in the given nesting area. Corrected inter-nest distance were necessary to compare the different nesting areas, as the size difference would cause an artefact. Namely, we can take only shorter distances on smaller areas compared to big areas. Therefore, statistically the distances are always smaller on a small area compared to a big one, regardless the nesting behaviour of the turtles. That is why corrected inter-nest distance is necessary in the comparison of the nesting areas. When the values is close to “1” the nests were located far from each other while the values close to “0” the nests were located closer to each other. We transformed the corrected inter-nest distance to square root to reach normal distribution. When we analysed data of the different nesting areas together, we used *glmer* with normal distribution with nesting areas as the random factor. We analysed the mean corrected inter-nest distance difference between nesting areas with *lm*. In the case of post-hoc multiply comparisons, we used Tukey-test (*glht*) from *multcomp* package [27]. We analysed the effects of years on the corrected inter-nest distance with *lm* and Tukey-test.

To compare the migration distances between the different nesting area and the lakes and investigate the yearly effects, we calculated the average migration distances (based on the distances between the nests and lakes per nesting areas) and the standard deviation of the migration distances based on the yearly data and the data independently of years.

We analysed the nest distance difference from the northern and southern pine forest, at pond 5 with an *lm.* We also used an *lm* to the analysis of the nest distance differences of the single clutch and the returning females from the northern pine forest. We tested whether there is an effect of years on the distance from the pine forest with an *lm* and a Tukey-test. To test the different distance from the northern pine forest between line and bunch-like nesters, and the effects of years on that we used *lm* and Tukey-test.

We tested the nest site fidelity by comparing the uncorrected inter-nest distances observed of the returning females to their previous nests and the inter-nest distances of single clutch females to each other. If the mean distance between nests of returning females is smaller than the mean inter-nest distances between all combination of single clutch females, then there is nest site fidelity [51, 71]. We compared the inter-nest distance of double clutching females to the inter-nest distance of returning females with an *lm*.

## Results

### Impact of habitat features on nesting attempts and successful hatchling emergence

The 91.61% of the nesting attempts occurred on the north side of the ponds (Fig. 2). In this way, nests, including ones on the inter-lake dams, were south-facing. The 8.39% of nesting attempts were on the south side of the ponds and on the dams between ponds. Nesting attempts were found in areas of bare or grassy sandy soils, which could be either large expanses of open sandy dry and semi-dry grassland associations (e.g. at pond 3–4, 5, 7, 8, 9, 10, 11) or small patches wedged into mosaic associations (habitat types e.g. scattered native trees on the east side of the pond 1‒2, pioneer shrub on the south part of the dam between pond 5–6 and scattered non-native trees on the damp between pond 7–8). The composition of habitat patches around the nesting area were very similar with a few exceptions (Fig. 2). Pond 1–2 was the most different from the other nesting areas. This pond was surrounded by a large area of deciduous forest, leaving only a small patch of open grassland immediately adjacent to the pond.

Analysing the effect of habitat types and environmental features on all successful nestings, the first axis explained 55.56% of the variance. The PCA showed that increased sunlight and slope had a positive weight on nest site selection of European pond turtle females. They selected mainly the sunny, downhill areas with the cover of closed sand steppes and uncharacteristic dry and semi-dry grasslands for nesting. Some vegetation types give positive effect in a mixture but negative effect as sole vegetation type, such as uncharacteristic grasslands and alien plants (black locust – *Robina pseudoacacia* and black pine – *Pinus nigra*). However, standing water had a negative effect (Table 1).

When we analysed the effect of habitat types and environmental features only on the protected nests, the first axis explained 83.89% of the variance. We found very similar patterns to the previous analysis, such as positive effect of closed sand steppes and uncharacteristic dry and semi-dry grasslands, wet and mesic pioneer scrubs occurring on sandy soil and the sunlight and the slope (Table 1).

If we analysed the effect of habitat types and environmental features only on the data of successful hatchling emergence, the first axis explained 91.88% of the variance. The pattern was very similar to the previous one, but the effect of wet and mesic shrubs disappeared as there was only one successful nest on this type. The emergence success was significantly higher in case if the score of the first PCA axis was higher (z = 2.26, *p* = 0.024, Additional file 1: Table S8), meaning that the increased sand soil cover, more sunlight and slope increases the emergence success. This effect was independent of the different nesting areas, as we got the exact same result with the mixed effect model. However, the extent of emergence success (how many percentages of hatchlings could emerge) was independent of the first axis (t = − 0.68, *p* = 0.501, Additional file 1: Table S9).

If the effect of the general environmental features of the nesting areas on the number of nesting attempts were analysed, there was no significant effect of slope (t = 1.93, *p* = 0.090), amount of sunlight (t = 0.97, *p* = 0.362) or exposure (t = 0.89, *p* = 0.400) on the number of all nesting attempts, when the data of the most preferred nest site (outlier) was excluded (Additional file 1: Table S10).

### Density of nesting attempts and comparison of inter-nest distances in nesting areas

The density of nesting attempts on all nesting areas showed no correlation with the increase in nesting area’s coverage (t = − 1.42, *p* = 0.183). If the exceptionally high density-value in 2015 (10 nests/10 m^2^) is excluded from the analysis (outlier), the increasing area significantly decreases the density value for successful attempts (t = − 2.71, *p* = 0.022) (Additional file 1: Table S11). Density values for successful nesting attempts showed greater year-to-year variability at the smaller nest site at pond 1–2 than at pond 5 (Additional file 1: Table S12).

**Fig. 3:**
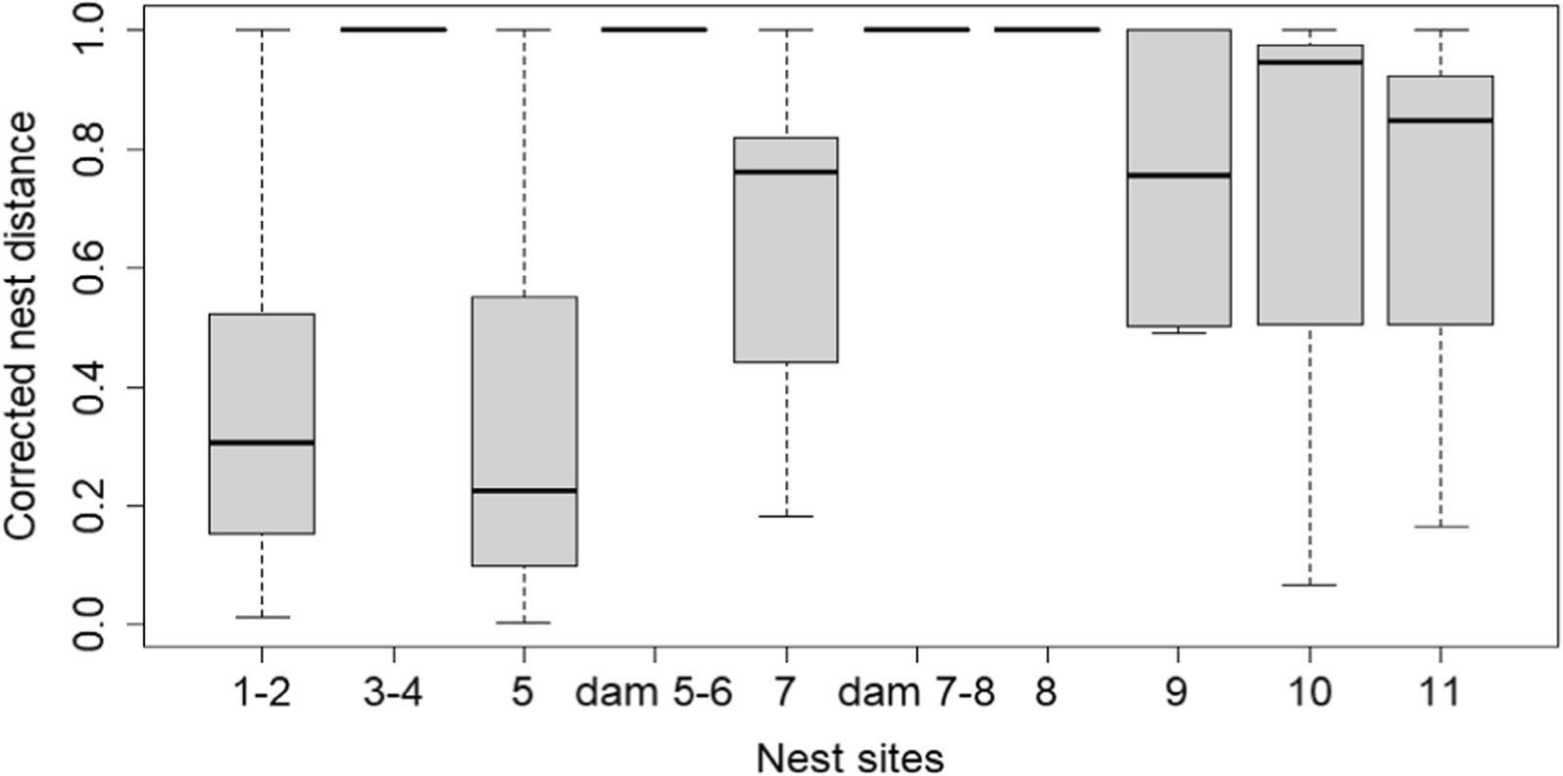
Corrected inter-nest distance (inter-nest distance divided by the longest inter-nest distance observed at the nesting area) of successful nesting attempts at the pond-system. The bottom and the top of the box are the first and third quartiles. The ends of the whiskers are the minimum and maximum excluding outliers

Whereas the distances between two successful nesting attempts were different at the 10 nesting areas and the sizes of the nesting areas were differ, we used the corrected inter-nest distance. Taking into account the coverage of nesting areas with analysing the corrected inter-nest distances of all nesting attempts, we obtained significant differences in the mean corrected distance only between nest site at pond 7 and pond 1–2 (t = − 4.19, *p* = 0.010) and pond 7 and pond 5 (t = − 4.14, *p* = 0.010) with higher values of distance at pond 7 (Fig. 3 and Additional file 1: Table S13).

**Fig. 4:**
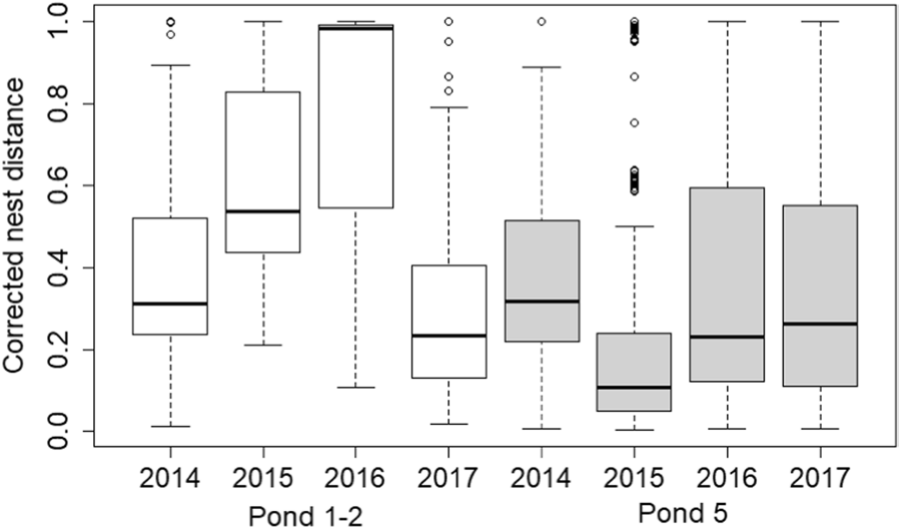
Changes of the corrected inter-nest distance (inter-nest distance divided by the longest inter-nest distance observed at the nesting area) between the successful nesting attempts at the two preferred nesting areas in the different years. Pond 1–2 boxes are white, Pond 5 boxes are grey. See boxplot description at Fig. 3. Open circles are outlier (more than 3/2 times of the upper or lower quartile)

For all nesting areas, the mean of the corrected inter-nest distance was smaller in 2015 than in 2014, while in 2016 and 2017 the corrected inter-nest distances are smaller than in 2015. We got similar results when we analysed only the two preferred nest sites. The mean value of the corrected inter-nest distance is smaller in 2015 than in 2014, while the mean of the corrected inter-nest distance is smaller in 2016 and 2017 than in 2015 (Fig. 4 and Additional file 1: Table S14).

### Nesting migration distances

European pond turtle had to migrate to reach the nesting area closest to the pond and find a suitable nest sites. The nesting migration distance was different from the ponds to the different nest sites (Table 2). The distances were generally the greatest at pond 5, which is the farthest from the ponds; we found most of the nests there. On the other hand, the other preferred nest site at pond 1–2 is one of the closest to the pond. The mean migratory distance (distances of nesting attempt from the shores of the nearest pond) was not different between years (Additional file 1: Table S15).

**Table 2:** Variation in the nesting migration distances to the sites of all nesting attempts at the nesting areas

Nesting areas	Number of all nesting attempts	Migration distance (m) from the pond to the sites of all nesting attempts		
		Mean	SD	Min.–max
Pond 1–2	31	6.24	3.36	0.82–16.58
Pond 3–4	5	16.25	3.14	13.26–21.55
Pond 5	87	98.73	11.05	76.71–122.38
Dam of ponds 5 and 6	4	16.79	7.40	8.19–26.24
Pond 7	7	43.39	11.30	27.27–55.76
Dam of ponds 7 and 8	3	3.82	0.38	3.54–4.26
Pond 8	5	43.05	2.80	38.34–45.40
Pond 9	5	50.90	5.72	41.09–55.54
Pond 10	5	31.76	13.42	16.17–50.52
Pond 11	3	62.83	39.21	34.53–107.59

### Nest site selection influenced by pine forest at pond 5

To the north, west and south of the most preferred nesting area at pond 5 there are pine forests (Fig. 2), which may partially shade the nests. We observed that the majority of all nesting attempts were made closer to the north than to the southern pine forest belt (t = − 15.35, *p* < 0.001). The 96.55% of the nests were located within 20 m from the edge of northern pine forest. The mean distance from the north pine forest to the double clutching nests and nests of returning females in the coming years were significantly closer (11.53 m) with a few meters (t = − 2.29, *p* = 0.023) than all nesting attempts of single nesting females (13.08 m) (Additional file 1: Tables S16 and S17). We found no effect of years on the variation in nest-pine forest distance.

Most nesting data were obtained from the nesting area at pond 5, bordered on three sides by pine forest. Here 14 females were observed nesting repeatedly. The position of nest sites of 5 females that returned more than two times and the other 9 returning females, which laid eggs two times had different nesting site selection patterns. In one case, 19 nests of 8 females were grouped in a small area at the western end of nesting area (in a bunch), bordered on three sides by pine forest. In the other case they were in a single line (15 nests of 6 female) bordered by pine forest to the north and south. In the case of the successful nesting attempts in a bunch, the nests were all close to each other in the western part of the nesting area, despite of the huge size of the areas. Among these bunch like nesting of returning females, one nested 4 times within an area of 424.50 m^2^, while another one 3 times within 73.50 m^2^. The mean distance of the bunch type nests from the edge of the north pine forest was 11.74 m (SD = 4.69 m, min.–max. = 5.79–23.86 m, N = 19), while the mean distance of the line type was 11.28 m (SD = 3.20 m, min.–max. = 3.89–14.69 m, N = 15). There was no significant difference in the mean nest distance from the edge of the north pine forest at the case of bunch and single line type nests (t = – 1.03, *p* = 0.309), and there was no difference in the mean distance between years (Additional file 1: Tables S17, S18 and S19).

### Nesting area and nest site fidelity

The 38 individually marked females did not choose another nesting area to lay eggs during the four years. This means that there is observable nesting area fidelity. We had 39 nesting data of 14 returning females to analyse the nesting area fidelity at the pond 1–2 and pond 5.

**Fig. 5:**
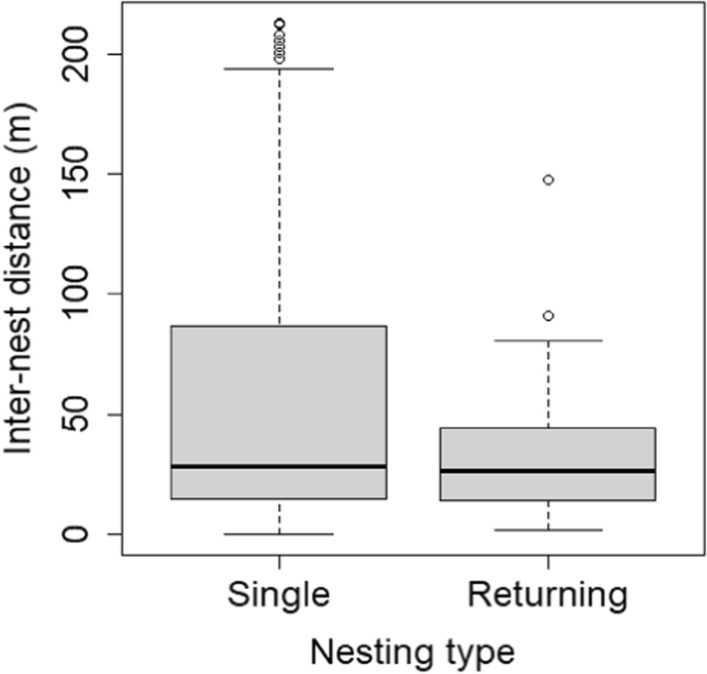
The difference in the inter-nest distances between all combination of “single clutch” females (observed laying once) and those returning more times (we gave distances compared to their first nests) at the two preferred nesting area. There was no significant difference between the groups. See boxplot description at Figs. 3 and 4

There was no significant difference (t = −  1.54, *p* = 0.126, Additional file 1: Table S20) compared to the mean inter-nest distances (m) between all combination of single clutch females calculated yearly and the mean of all inter-nest distances of returning females (measured always from their first known nest). We hypothesised that nest site fidelity would be better expressed if we use the mean of the new nesting distances from the known first nest site, rather than the mean distance between all their nests. But the analysis in this case also showed no significant difference between inter-nest distances (t = −  1.40, *p* = 0.162) (Fig. 5 and Additional file 1: Table S21). The only significant difference between years was in 2017, when the distances between nests of single-clutch females are larger than those of returning females (t = 3.16, *p* = 0.002, Additional file 1: Table S22).

**Fig. 6:**
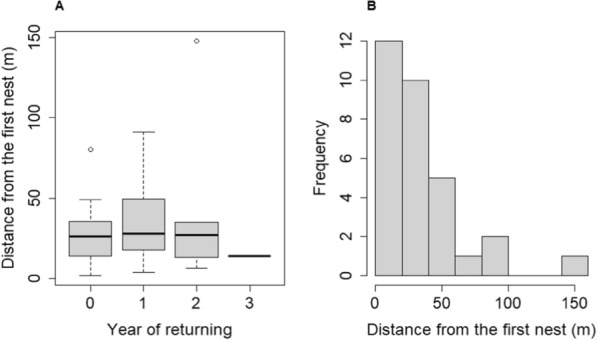
A: Distance between nest sites of a particular female in relation to their first nest at the two preferred nesting area in consecutive years. (0 = double clutching in the same year, 1–3 = 1–3 years returning after the first nesting). There was no significant difference between the groups. B: The histogram of distances (m) between nests of returning females relative to the first nests. See boxplot description at Figs. 3 and 4

When we compared the distances (m) between new nest sites of returning females to their first nest site within the year (0), and after 1–2–3 years, we found no significant difference (1st year returning: t = 0.42, *p* = 0.676; 2nd year returning: t = 0.97, *p* = 0.339; 3rd year returning: t = −  0.44, *p* = 0.661) (Fig. 6A and Additional file 1: Table S23). The frequency distribution of the new nest sites distances of returning females from their first nests shows that in most of cases they are within 20‒40 m (Fig. 6B).

## Discussion

### Impact of habitat features on nesting attempts, nest densities and inter-nest distances

Nesting attempts were found in areas of bare or grassy sandy soils, covered mostly by dry and semi-dry grassland associations or small patches wedged into mosaic associations in the Babat Valley system. Most of the nesting attempts were south-facing, occurred on the north side of the ponds. We found preference for sandy soil for nesting as we found positive weights on sand steppes and black pine plantations (historically, it was planted on sandy soil in Hungary) in the PCA, as well-supported by the literature [49, 60, 77, 79]. We assume that the negative weight of the uncharacteristic dry grassland was caused by the too many weed and alien species that made these areas unsuitable for nesting. The positive effect of black pine plantations on nest site selection, may due to their shading effect, which increases the security of the migration route and provide the opportunity to ensure optimal thermal conditions for incubation [10, 20, 71].

We also found positive weights on the shrub vegetation for egg-laying, however, we found only one successful nest here. Zuffi and Rovina [79] found also evidence that nests were selected in sunny bushy areas. It is possible that turtles select these sites based on the amount of sunlight and the loose-soil [39]. We found that they often lay their eggs in agricultural areas, on the edges of agricultural fields, on field roads, or on road shoulders, mentioned by Kosinski [31], Mitrus [46] also. In these areas turtles are potentially vulnerable to road associated mortality [67].

We found that the increase of sandy soil cover, more sunlight and increased slope increased the emergence success of hatchlings but was independent of the first axis of the environmental factors, so most probably this rather depends on environmental factors not included in the survey, or intrinsic factors. These environmental factors provide the optimal hatching conditions [43, 65]. Our PCA showed a negative weight on standing water, what we founded at the most preferred nesting area at pond 5 in Babat Valley, which is the farthest from the pond. Mitrus [47] support this result, it is possible that the microclimatic conditions are better farther from the pond, maybe a little warmer and the soil is less moist. In contrast, Liuzzo et al. [36] found that sun exposure and sandy soil with low organic matter content had a negative effect on incubation conditions, while plant cover and a nest site closer to water had a positive effect.

We found that density of successful nesting attempts taking into account all nest sites showed no correlation with the increase in nesting area’s coverage, but excluded one outlier the increasing area significantly decreases the density. This means that there are more or less sufficient nest sites near almost all lakes in the Babat Valley.

Although, the mean distances between nesting attempts varied between lakes, when size of nesting area were taken into account and used the corrected inter-nest distance, there were only a few nesting areas with significant differences in the corrected inter-nest distance, such as the difference between pond 7 and the two preferred nesting areas. This may be explained by the fact that nests were laid in the edge of the relatively small area on the nesting area at pond 7, spaced apart because the central part of the area was unsuitable for egg-laying.

### Nesting migration distances

In our survey, the maximum nesting migration distance to the most preferred nesting area at pond 5 was found to be 122.8 m. Nesting almost at the edge of the pond was found at the other preferred nesting area at pond 1–2, where the closest nest to the water was only 0.82 m away and the farthest was 16.58 m away. Cadi et al. [6], Escoriza et al. [16], Bona et al. [5] and Novotny et al. [55] reported nests directly next or very close to the water's shore typically up to a few hundred meters. However, in a few cases it can happen that migration exceeds a kilometre [44, 48], or seldom exceed 1.5 km [32, 34].

In our study, we found that during their nesting migration, turtles passed through reed beds, scrub strips, deciduous or coniferous forests, and sections with buildings to reach the nearest grassland on the northern side of the ponds. Studies revealed that the selected nest sites were mostly located near forested areas [20, 28, 42, 65], which may provide various advantages to turtles in migration as well. The presence of a canopy shade reduces the risk of desiccation, optimizing sun exposure, the little or no undergrowth facilitates movement, and the presence of litter helps to provide a hiding place. The presence of woodland is also a major advantage for hatchlings in reaching the water safely [19, 23, 54].

### Effect of pine forest on nest site selection

In the most preferred nesting area at pond 5, turtles laid their eggs at average 12.47 ± 4.57 m from the edge of the northern pine forest on a sandy loam grassland. The double clutching and returning females may have more experience to select the right nest site because their nests were significantly closer (11.53 m) to the edge of the pine forest than those of the single clutching females. The nest distances from the pine forest did not show any significant variation over the years, indicating that a certain degree of stability of this microhabitat selection can be observed. Liuzzo et al. [36] also found that plant cover and canopy cover had a beneficial effect on the microclimate of nesting sites for *E. orbicularis*. Christie et al. [10] and St. John [69] suggest patterns of nest site philopatry in *E. marmorata*, with several females returning to nest in close proximity to previous nesting sites in consecutive years. St. John and Geist [70] and St. John [71] identified preference for nesting within 10–15 m from the edge of the tree line of *E. marmorata* and hypothesized that these distances would provide the optimal thermal conditions for incubation by alternating exposure to full sun and shade throughout the day.

Based on the two nesting site distribution pattern (bunch and line type) of returning females there was no significant difference when comparing the nest distances from pine forest, indicating that despite their different use of the area, they almost exclusively chose the strip near the northern pine forest.

### Nesting area and nesting site fidelity

We did not observe nesting area shifts based on marked and recaptured individuals in the Babat lake system. This means strong nesting area fidelity. This could mean that in the Babat Valley, almost all ponds have sufficient nesting areas nearby, so there is no need to visit another area. In the case of Pond 1–2, the turtles have no opportunity to cross to other ponds because of the highway that runs through the valley. However, the other nesting areas had no barrier separating them from each other.

In our study we found that females nested mostly within 20 and 40 m compared to their first nest site and the inter-nest distances measured in consecutive years. Several studies have shown that there is a tendency among freshwater turtles, including *E. orbicularis* females to return to the same or nearby nest site over a long period sometimes to several dozen years [5, 6, 13, 28, 43, 46, 47, 52, 53, 55, 69, 72, 80].

We did not find clear evidence of nest site fidelity as there was no statistical difference between the inter-nest distance of the returning females and the single nest females. St. John [71] found that mean inter-nest distances of *E. marmorata* indicates a tendency that the inter-nest distance between consecutive nests of individual females is smaller than the mean inter-nest distance of females in the whole population. Similar results were shown in *Chrysemys picta* [33, 61] and in *Graptemys geographica* [51]. According to St. John et al. [69] and St. John [72], only a part of females show nesting site fidelity, with the rest laying their nests further apart to adapt to the changing environment.

Other researchers state that the increase in the distance between consecutive nestings may indicate that females are actively searching for the most suitable areas for nests, and are able to respond flexibly to changing environmental conditions [5, 46, 49, 51, 53]. We did not observed changes of habitat parameter that would have influenced either nesting area or nest site selection during our 4-years survey.

## Conclusions

European pond turtle females may have to migrate up to several hundred metres to reach the nearest most suitable sunny, sloping sandy soil and sparse grassland for nesting. Forests crossing the migration route are not only an overcoming barrier, but also a safer way to migrate. Females lay their eggs at a selected distance from the forest edges that provides the right incubation soil temperature through shading. We have shown that the degradation of open steppe vegetation (shrub encroachment), occurrence of weeds, invasive and disturbance tolerant species have a negative effect on the selection of nesting sites. This may also mean that the new nesting site of the same female may located further away from the previous one. Based on the nesting patterns of individually identified females in a given year and in subsequent years, we found a strict nesting area choice, while there was no clear nest site fidelity. The wetland and terrestrial habitat protection and the maintenance of mosaic habitat structure should be basic priorities in European pond turtle conservation programs. If necessary, the succession process should be slowed or stopped by removing shrubs and trees from the actual nesting area.

Based on our set of information we could suggest a spatial and temporal scheduling of land management and agricultural work to the local farmers. Although these are adapted to local specific circumstances, several elements are considered to be generally applicable. The first step in conservation measures is to locate and map the nesting area, e.g. by identifying the sites of nests depredated by predators in the previous year. If the turtle’s nesting area is in the grass edge zone of agricultural area, the area must be clearly marked in order to ensure that agricultural machinery is not used to access to the field or to turn around just there. This solution is easily accepted by farmers and usually does not cause conflict. If the actual nesting area is within an agricultural area, the boundaries of the area must be clearly marked for machine handlers also. Since the nesting areas’ sizes are usually not significant compared to the cultivated fields, the conflict can be avoided or reduced by establishing an appropriate relationship with the farmer and explaining the importance of conservation objectives. All work (ploughing, tilling, sowing, plant care, harvesting) that would disturb eggs or overwintering hatchlings in the nest on this marked nesting area should be avoided throughout the year. Agricultural machinery should avoid the migration routes of adult turtles and emerged hatchlings during the concerned period. This is possible without problems on the available alternative bypass roads if there are. In the absence of this, an alternative route, which is acceptable, should be defined with the farmer. Adult turtles migrate from early May to mid-July, hatchlings emerge in a current year from mid-August to the end of September, while overwintered hatchlings from mid-March to end-April. The protection of overwintering hatchlings is very important, as their numbers can reach up to 40% of the total number of hatchlings [29]. These spatial and temporal constraints can only be achieved by upkeeping constant contact with farmers and passing on information, so that the farmer's potential losses can be minimised.

Besides agricultural activities, the presence of predators has the greatest impact on the reproductive success and survival of the population. Under strong predation pressure, successful reproduction of the population can be ensured by predator control or by using metal grids, what is a relatively time consuming but most effective nest protection [29]. Our experience shows that the loss due to predators is very high when nests are not protected. This nest protection should apply to both natural habitats and agricultural lands.

### Supplementary Information

Additional file 1. Summary data tables and statistical tables.

## Data Availability

The data supporting this article will be available upon request.
