# Supplementary material for: Nest site selection and fidelity of European pond turtle (Emys orbicularis) population of Babat Valley (Gödöllő, Hungary)
Source: Front Zool. 2024 Aug 12;21:20. doi: 10.1186/s12983-024-00541-3 (PMC11318129; doi:10.1186/s12983-024-00541-3)
Supplement: Supplementary file 1 — Additional file 1. Summary data tables and statistical tables. [file 12983_2024_541_MOESM1_ESM.docx]

**Frontiers in Zoology**

**Nest site selection** **and fidelity** **of European pond turtle (*Emys orbicularis*) population of Babat Valley (Gödöllő, Hungary)**

**István Kiss ^a^, Gergő Erdélyi ^a^, Borbála Szabó ^b *^**

^a^ Hungarian University of Agriculture and Life Sciences, Department of Zoology and Animal Ecology, Páter K. street 1., Gödöllő, H–2103, Hungary

^b^ University of Bremen, UFT, General and Theoretical Ecology, Leobener Str. 6, 28359, Bremen, Germany

*Corresponding Author: Borbála Szabó, [bszabo@uni-bremen.de](mailto:szbori001@gmail.com)

ORCID numbers:

István Kiss: 0000-0002-0821-8667

Gergő Erdélyi: 0009-0008-4250-1758

Borbála Szabó^:^ 0000-0001-7587-1597

**Table S1.** Number of nesting attempts and the emergence success at the different nesting areas.

| Nesting areas | Number of unsuccessful nesting | Number of depredated nests | Number of protected nests | Number of nests with emergence | Emergence success (%) of hatchlings |
| --- | --- | --- | --- | --- | --- |
| pond 1–2 | 6 | 18 | 7 | 4 | 75.56 |
| pond 3–4 | 1 | 4 | 0 | 0 | 0 |
| pond 5 | 13 | 32 | 42 | 35 | 90.88 |
| dam of ponds 5 and 6 | 2 | 1 | 1 | 1 | 100.00 |
| pond 7 | 0 | 7 | 0 | 0 | 0 |
| dam of ponds 7 and 8 | 1 | 2 | 0 | 0 | 0 |
| pond 8 | 0 | 5 | 0 | 0 | 0 |
| pond 9 | 0 | 5 | 0 | 0 | 0 |
| pond 10 | 0 | 5 | 0 | 0 | 0 |
| pond 11 | 0 | 3 | 0 | 0 | 0 |

**Table S2.** Number of surveys during the nesting season at the nesting areas.

| Years | Pond 1-2 | Pond 5 | All other ponds per pond |
| --- | --- | --- | --- |
| 2014 | 20 | 20 | - |
| 2015 | 39 | 39 | 7 |
| 2016 | 24 | 24 | 7 |
| 2017 | 20 | 20 | 4 |
| 2014-2017 | 103 | 103 | 18 |

|  | B1a.B5 | H5b.OC | J5 | K2 | OC | P2a | RA | RA.B1a.B5 | RB | RC | RDb | S1 | S1.S4 | S4 |
| --- | --- | --- | --- | --- | --- | --- | --- | --- | --- | --- | --- | --- | --- | --- |
| B1a.B5 | 1 | -0.1409 | -0.0392 | 0.3430 | 0.2370 | 0.1279 | 0.1436 | 0.3788 | -0.0919 | -0.0392 | -0.2062 | -0.1033 | -0.1841 | -0.3754 |
| H5b.OC | -0.1409 | 1 | 0.1162 | -0.1444 | -0.7512 | 0.3435 | -0.5506 | -0.0349 | -0.3219 | 0.1162 | -0.5711 | -0.6243 | 0.2450 | 0.8157 |
| J5 | -0.0392 | 0.1162 | 1 | -0.0101 | -0.0542 | -0.0718 | 0.0773 | -0.0125 | -0.0226 | 1 | -0.0401 | -0.0445 | -0.0362 | -0.1069 |
| K2 | 0.3430 | -0.1444 | -0.0101 | 1 | 0.0460 | 0.4364 | -0.0715 | -0.0166 | -0.0217 | -0.0101 | -0.0533 | -0.0551 | -0.0481 | -0.0166 |
| OC | 0.2370 | -0.7512 | -0.0542 | 0.0460 | 1 | -0.1805 | 0.1408 | -0.0455 | 0.1269 | -0.0542 | 0.1317 | 0.2412 | -0.2500 | -0.6759 |
| P2a | 0.1279 | 0.3435 | -0.0718 | 0.4364 | -0.1805 | 1 | -0.5060 | -0.1176 | -0.1844 | -0.0718 | -0.3771 | -0.3235 | -0.2075 | 0.4676 |
| RA | 0.1436 | -0.5506 | 0.0773 | -0.0715 | 0.1408 | -0.5060 | 1 | 0.0588 | 0.5102 | 0.0773 | 0.7463 | 0.6882 | -0.2497 | -0.7032 |
| RA.B1a.B5 | 0.3788 | -0.0349 | -0.0125 | -0.0166 | -0.0455 | -0.1176 | 0.0588 | 1 | -0.0370 | -0.0125 | -0.0657 | -0.0077 | -0.0594 | -0.1752 |
| RB | -0.0919 | -0.3219 | -0.0226 | -0.0217 | 0.1269 | -0.1844 | 0.5102 | -0.0370 | 1 | -0.0226 | 0.6402 | 0.2572 | -0.1073 | -0.3167 |
| RC | -0.0392 | 0.1162 | 1 | -0.0101 | -0.0542 | -0.0718 | 0.0773 | -0.0125 | -0.0226 | 1 | -0.0401 | -0.0445 | -0.0362 | -0.1069 |
| RDb | -0.2062 | -0.5711 | -0.0401 | -0.0533 | 0.1317 | -0.3771 | 0.7463 | -0.0657 | 0.6402 | -0.0401 | 1 | 0.8254 | -0.1904 | -0.5618 |
| S1 | -0.1033 | -0.6243 | -0.0445 | -0.0551 | 0.2412 | -0.3235 | 0.6882 | -0.0077 | 0.2572 | -0.0445 | 0.8254 | 1 | -0.2114 | -0.6235 |
| S1.S4 | -0.1841 | 0.2450 | -0.0362 | -0.0481 | -0.2500 | -0.2075 | -0.2497 | -0.0594 | -0.1073 | -0.0362 | -0.1904 | -0.2114 | 1 | 0.3859 |
| S4 | -0.3754 | 0.8157 | -0.1069 | -0.0166 | -0.6759 | 0.4676 | -0.7032 | -0.1752 | -0.3167 | -0.1069 | -0.5618 | -0.6235 | 0.3859 | 1 |
| S7 | 0.2822 | -0.4437 | -0.0312 | -0.0414 | 0.4659 | -0.2930 | -0.0504 | -0.0077 | -0.0922 | -0.0312 | -0.1638 | -0.1818 | -0.1401 | -0.3620 |
| T1 | -0.0170 | -0.4655 | -0.0329 | -0.0437 | 0.6655 | -0.3091 | -0.0599 | 0.0504 | -0.0974 | -0.0329 | -0.1728 | -0.1905 | -0.1466 | -0.3825 |
| T10 | 0.6132 | 0.1815 | 0.3526 | -0.0303 | -0.1209 | -0.2142 | 0.3343 | 0.3511 | -0.0675 | 0.3526 | -0.1198 | -0.1180 | -0.1082 | -0.3192 |
| U11 | 0.3124 | -0.2181 | -0.0153 | 0.0111 | 0.5694 | 0.2362 | -0.1032 | -0.0251 | 0.0792 | -0.0153 | -0.0759 | 0.1441 | -0.0727 | -0.2135 |
| U4 | 0.1088 | -0.3895 | -0.0274 | -0.0363 | 0.4927 | -0.2572 | -0.1722 | 0.0411 | -0.0810 | -0.0274 | -0.1438 | -0.1596 | -0.1299 | -0.3832 |
| U9 | 0.0652 | -0.9032 | -0.0648 | 0.0484 | 0.6280 | -0.3787 | 0.6548 | -0.0399 | 0.3683 | -0.0648 | 0.7236 | 0.8337 | -0.3047 | -0.8545 |
| Distance to Lake | -0.3876 | 0.8535 | 0.0889 | -0.1261 | -0.5763 | 0.2670 | -0.7346 | -0.1477 | -0.3690 | 0.0889 | -0.6731 | -0.7365 | 0.4424 | 0.9216 |
| Sunlight | -0.1462 | 0.9133 | 0.0641 | -0.1858 | -0.6204 | 0.2786 | -0.6286 | 0.0769 | -0.4143 | 0.0641 | -0.7351 | -0.8019 | 0.2999 | 0.8188 |
| Slope | -0.3211 | 0.8665 | -0.0321 | -0.1780 | -0.5932 | 0.3412 | -0.7288 | -0.0526 | -0.4013 | -0.0321 | -0.7121 | -0.7834 | 0.3337 | 0.9176 |

**Table S3.** Pearson correlation matrix for environmental parameters for all nesting attempts in their 100m radius. Table continued on the next page.

The vegetation parameters are shortened as follows: **B1a.B5**: Eu- and mesotrophic reed and Typha beds mixed with tussock sedge communities, **H5b.OC**: Closed sand steppes mixed with uncharacteristic dry and semi-dry grasslands, **J5**: Riverine oak-elm-ash woodlands, **K2**: Sessile oak-hornbeam woodlands, **OC**: Uncharacteristic dry and semi-dry grasslands, **P2a:** Wet and mesic pioneer scrub, **RA**: Scattered native trees or narrow tree lines, **RA.B1a.B5**: mixture of trees, reed and tussock sedge, **RB**: Uncharacteristic or pioneer softwood forests, **RC**: Uncharacteristic hardwood forests and plantations, **RDb:** Non-native deciduous forests and plantations mixed with native tree species, **S1:** *Robinia pseudoacacia* plantations, **S1.S4**: *Robinia pseudoacacia* plantations mixed with scots and black pine plantations, **S4:** Scots and black pine plantations, **S7:** Scattered trees or narrow tree lines of non-natives tree species, **T1:** Annual intesive arable fields, **T10:** New abandonments of arable lands, **U11**: Farms, **U4:** Yards, wastelands, dumping grounds, **U9:** Standing waters

**Table 3.** Continued

|  | S7 | T1 | T10 | U11 | U4 | U9 | Distance to Lake | Sunlight | Slope |
| --- | --- | --- | --- | --- | --- | --- | --- | --- | --- |
| B1a.B5 | 0.2822 | -0.0170 | 0.6132 | 0.3124 | 0.1088 | 0.0652 | -0.3876 | -0.1462 | -0.3211 |
| H5b.OC | -0.4437 | -0.4655 | 0.1815 | -0.2181 | -0.3895 | -0.9032 | 0.8535 | 0.9133 | 0.8665 |
| J5 | -0.0312 | -0.0329 | 0.3526 | -0.0153 | -0.0274 | -0.0648 | 0.0889 | 0.0641 | -0.0321 |
| K2 | -0.0414 | -0.0437 | -0.0303 | 0.0111 | -0.0363 | 0.0484 | -0.1261 | -0.1858 | -0.1780 |
| OC | 0.4659 | 0.6655 | -0.1209 | 0.5694 | 0.4927 | 0.6280 | -0.5763 | -0.6204 | -0.5932 |
| P2a | -0.2930 | -0.3091 | -0.2142 | 0.2362 | -0.2572 | -0.3787 | 0.2670 | 0.2786 | 0.3412 |
| RA | -0.0504 | -0.0599 | 0.3343 | -0.1032 | -0.1722 | 0.6548 | -0.7346 | -0.6286 | -0.7288 |
| RA.B1a.B5 | -0.0077 | 0.0504 | 0.3511 | -0.0251 | 0.0411 | -0.0399 | -0.1477 | 0.0769 | -0.0526 |
| RB | -0.0922 | -0.0974 | -0.0675 | 0.0792 | -0.0810 | 0.3683 | -0.3690 | -0.4143 | -0.4013 |
| RC | -0.0312 | -0.0329 | 0.3526 | -0.0153 | -0.0274 | -0.0648 | 0.0889 | 0.0641 | -0.0321 |
| RDb | -0.1638 | -0.1728 | -0.1198 | -0.0759 | -0.1438 | 0.7236 | -0.6731 | -0.7351 | -0.7121 |
| S1 | -0.1818 | -0.1905 | -0.1180 | 0.1441 | -0.1596 | 0.8337 | -0.7365 | -0.8019 | -0.7834 |
| S1.S4 | -0.1401 | -0.1466 | -0.1082 | -0.0727 | -0.1299 | -0.3047 | 0.4424 | 0.2999 | 0.3337 |
| S4 | -0.3620 | -0.3825 | -0.3192 | -0.2135 | -0.3832 | -0.8545 | 0.9216 | 0.8188 | 0.9176 |
| S7 | 1 | 0.6215 | -0.0182 | -0.0625 | 0.7989 | 0.2434 | -0.2768 | -0.2640 | -0.2420 |
| T1 | 0.6215 | 1 | -0.0943 | -0.0660 | 0.6551 | 0.2202 | -0.1808 | -0.1612 | -0.1383 |
| T10 | -0.0182 | -0.0943 | 1 | -0.0457 | 0.0125 | -0.1271 | -0.1466 | 0.1631 | -0.1103 |
| U11 | -0.0625 | -0.0660 | -0.0457 | 1 | -0.0549 | 0.2405 | -0.2099 | -0.2808 | -0.2720 |
| U4 | 0.7989 | 0.6551 | 0.0125 | -0.0549 | 1 | 0.2760 | -0.2301 | -0.21651 | -0.2046 |
| U9 | 0.2434 | 0.2202 | -0.1271 | 0.2405 | 0.2760 | 1 | -0.9039 | -0.9665 | -0.9385 |
| Distance to Lake | -0.2768 | -0.1808 | -0.1466 | -0.2099 | -0.2301 | -0.9039 | 1 | 0.9058 | 0.9575 |
| Sunlight | -0.2640 | -0.1612 | 0.1631 | -0.2808 | -0.2165 | -0.9665 | 0.9058 | 1 | 0.9608 |
| Slope | -0.2420 | -0.1383 | -0.1103 | -0.2720 | -0.2046 | -0.9385 | 0.9575 | 0.9608 | 1 |

**Table S4**. Pearson’s correlation matrix for PCA of nesting success in 100m radius, See description of vegetation type codes at Table S3.

|  | B1a.B5 | H5b.OC | K2 | OC | P2a | RA | RB | RDb | S1 | S1..S4 | S4 | U9 | Distance to Lake | Sunlight | Slope |
| --- | --- | --- | --- | --- | --- | --- | --- | --- | --- | --- | --- | --- | --- | --- | --- |
| B1a.B5 | 1 | -0.2830 | 0.9785 | 0.2247 | 0.6346 | -0.1020 | -0.0669 | -0.1001 | -0.1014 | -0.1256 | -0.0888 | 0.0677 | -0.2330 | -0.2788 | -0.2788 |
| H5b.OC | -0.2830 | 1 | -0.3201 | -0.9816 | 0.2322 | -0.9062 | -0.5945 | -0.8886 | -0.9003 | 0.1193 | 0.9533 | -0.9571 | 0.9481 | 0.9833 | 0.9833 |
| K2 | 0.9785 | -0.3201 | 1 | 0.2709 | 0.5341 | -0.0615 | -0.0404 | -0.0604 | -0.0611 | -0.0758 | -0.1201 | 0.1105 | -0.2379 | -0.3255 | -0.3255 |
| OC | 0.2247 | -0.9816 | 0.2709 | 1 | -0.2771 | 0.9420 | 0.6179 | 0.9237 | 0.9359 | -0.2324 | -0.9839 | 0.9840 | -0.9584 | -0.9984 | -0.9984 |
| P2a | 0.6346 | 0.2322 | 0.5341 | -0.2771 | 1 | -0.4692 | -0.3078 | -0.4601 | -0.4662 | -0.4234 | 0.3060 | -0.3735 | 0.0799 | 0.2404 | 0.2404 |
| RA | -0.1020 | -0.9062 | -0.0615 | 0.9420 | -0.4692 | 1 | 0.6752 | 0.9770 | 0.9849 | -0.2146 | -0.9769 | 0.9816 | -0.9107 | -0.9216 | -0.9216 |
| RB | -0.0669 | -0.5945 | -0.0404 | 0.6179 | -0.3078 | 0.6752 | 1 | 0.7820 | 0.5509 | -0.1407 | -0.6408 | 0.6021 | -0.6026 | -0.6046 | -0.6046 |
| RDb | -0.1001 | -0.8886 | -0.0604 | 0.9237 | -0.4601 | 0.9770 | 0.7820 | 1 | 0.9458 | -0.2104 | -0.9579 | 0.9471 | -0.8943 | -0.9038 | -0.9038 |
| S1 | -0.1014 | -0.9003 | -0.0611 | 0.9359 | -0.4662 | 0.9849 | 0.5509 | 0.9458 | 1 | -0.2132 | -0.9706 | 0.9805 | -0.9030 | -0.9157 | -0.9157 |
| S1.S4 | -0.1256 | 0.1193 | -0.0758 | -0.2324 | -0.4234 | -0.2146 | -0.1407 | -0.2104 | -0.2132 | 1 | 0.2938 | -0.2274 | 0.3858 | 0.2328 | 0.2328 |
| S4 | -0.0888 | 0.9533 | -0.1201 | -0.9839 | 0.3060 | -0.9769 | -0.6408 | -0.9579 | -0.9706 | 0.2938 | 1 | -0.9931 | 0.9696 | 0.9736 | 0.9736 |
| U9 | 0.0677 | -0.9571 | 0.1105 | 0.9840 | -0.3735 | 0.9816 | 0.6021 | 0.9471 | 0.9805 | -0.2274 | -0.9931 | 1 | -0.9474 | -0.9731 | -0.9731 |
| Distance to Lake | -0.2330 | 0.9481 | -0.2379 | -0.9584 | 0.0799 | -0.9107 | -0.6026 | -0.8943 | -0.9030 | 0.3858 | 0.9696 | -0.9474 | 1 | 0.9556 | 0.9556 |
| Sunlight | -0.2788 | 0.9833 | -0.3255 | -0.9984 | 0.2404 | -0.9216 | -0.6046 | -0.9038 | -0.9157 | 0.2328 | 0.9736 | -0.9731 | 0.9556 | 1 | 1 |
| Slope | -0.2788 | 0.9833 | -0.3255 | -0.9984 | 0.2404 | -0.9216 | -0.6046 | -0.9038 | -0.9157 | 0.2328 | 0.9736 | -0.9731 | 0.9556 | 1 | 1 |

**Table S5**. Pearson’s correlation matrix for nesting attempt numbers and basic environmental parameters of the nesting areas

|  | No. Nest | Nesting area size | Distance from the lake | Sunlight | Slope | Exposure |
| --- | --- | --- | --- | --- | --- | --- |
| No. Nest | 1 | 0.6500 | 0.7448 | 0.3235 | 0.5626 | 0.2997 |
| Nesting area size | 0.6500 | 1 | 0.5829 | 0.7542 | 0.6309 | 0.551 |
| Distance from the lake | 0.7448 | 0.5829 | 1 | 0.7314 | 0.9308 | 0.5366 |
| Sunlight | 0.3235 | 0.7542 | 0.7314 | 1 | 0.8837 | 0.7093 |
| Slope | 0.5626 | 0.6309 | 0.9308 | 0.8837 | 1 | 0.7157 |
| Exposure | 0.2997 | 0.551 | 0.5366 | 0.7093 | 0.7157 | 1 |

**Table S6.** Pearson’s correlation matrix for nest density table

|  | Area | No. Nest | Nest density |
| --- | --- | --- | --- |
| Area | 1 | 0.6140 | -0.4867 |
| No. Nest | 0.6140 | 1 | -0.3192 |
| Nest density | -0.4867 | -0.3192 | 1 |

**Table S7.** Inter-nest distances between successful nesting attempts at the nesting areas, by analysing four years (2014–2017) of data together. Where there were only two successful nests, the distance between them is included in the min.–max. values, and the mean, median and standard deviation values are not meaningful (NA).

| Nesting area | Number of successful nestings | Inter-nest distances between successful nesting attempts | | | |
| --- | --- | --- | --- | --- | --- |
|  |  | Min.–max. (m) | Median (m) | Mean (m) | SD |
| Pond 1–2 | 25 | 0.00–160.26 | 18.36 | 31.46 | 36.59 |
| Pond 3–4 | 4 | 6.71–32.70 | 19.70 | 19.70 | 18.38 |
| Pond 5 | 74 | 0.00–214.34 | 34.00 | 59.10 | 56.52 |
| Dam of ponds 5 and 6 | 2 | 20.10 | NA | NA | NA |
| Pond 7 | 7 | 3.16–17.26 | 13.15 | 11.08 | 4.74 |
| Dam of ponds 7 and 8 | 2 | 69.30 | NA | NA | NA |
| Pond 8 | 5 | 24.70–38.21 | 31.46 | 31.46 | 9.55 |
| Pond 9 | 5 | 14.42–69.36 | 34.74 | 38.31 | 22.81 |
| Pond 10 | 5 | 15.03–224.33 | 212.18 | 150.51 | 117.49 |
| Pond 11 | 3 | 39.32–236.66 | 200.35 | 158.77 | 105.03 |

**Table S8.** Full model output of effect of PCA axis on emergence success with *glm* and *glmer* with binomial distribution. We got identical results for both; therefore, we describe the results only once.

|  | Estimate | Std. Error | z-value | p-value |
| --- | --- | --- | --- | --- |
| intercept | 0.4054 | 0.7280 | 0.56 | 0.578 |
| axis1 | 0.0292 | 0.0129 | 2.26 | 0.024 |

**Tables S9**. Full model output of the effect of PCA axis on the extent of emergence success with *lm* and *glmer* with normal distribution. We got identical results for both, therefore we describe the results only once.

|  | Estimate | Std. Error | t-value | p-value |
| --- | --- | --- | --- | --- |
| intercept | 98.1613 | 7.8323 | 12.53 | <0.001 |
| axis1 | -0.0785 | 0.1155 | – 0.68 | 0.501 |

**Table S10.** Full model outputs of effect of general environmental parameters of the nesting area on the number of nesting attempts. First three *lm* models are on the full data, the second three is without the outlier pond 5.

|  | Estimate | Std. Error | t-value | p-value |
| --- | --- | --- | --- | --- |
| Full data |  |  |  |  |
| intercept | -22.024 | 20.831 | -1.06 | 0.321 |
| slope | 2.207 | 1.147 | 1.93 | 0.090 |
| intercept | -71.5652 | 90.4346 | -0.79 | 0.452 |
| sunlight | 0.9783 | 1.0117 | 0.97 | 0.362 |
| intercept | 4.00 | 15.47 | 0.26 | 0.802 |
| exposure | 16.43 | 18.49 | 0.89 | 0.400 |
| Without pond 5 | |  |  |  |
| intercept | 16.5000 | 9.7695 | 1.69 | 0.135 |
| slope | -0.5750 | 0.5983 | -0.96 | 0.369 |
| intercept | 38.9800 | 33.4132 | 1.17 | 0.282 |
| sunlight | -0.3580 | 0.3791 | -0.94 | 0.376 |
| intercept | 4 | 15.47 | 0.259 | 0.802 |
| exposure | 16.43 | 18.49 | 0.889 | 0.400 |

**Table S11.** Full model outputs of effect the size of nesting area on the density of the nesting attempts. Both are *lm* models. The first one is with the total dataset, the second one is without the outlier value from 2015.

|  | Estimate | Std. Error | t-value | p-value |
| --- | --- | --- | --- | --- |
| Full data |  |  |  |  |
| intercept | 2.1933 | 1.0167 | 2.16 | 0.054 |
| nesting area | -0.0018 | 0.0013 | – 1.42 | 0.183 |
| Without outlier 2015 | | | | |
| intercept | 0.7743 | 0.1700 | 4.56 | 0.001 |
| nesting area | -0.0005 | 0.0002 | – 2.71 | 0.022 |

**Table S12.** Yearly variation of nest density in the case of all nesting attempts and highlighted of successful nesting attempts in association with the sizes of the actual nesting areas. NA means: the number of nesting attempts observed in a given year were zero or if was 1, 2, the density values were not meaningful, not calculated.

| Nesting area | Year | All nesting attempts | | | Successful nesting attempts | | |
| --- | --- | --- | --- | --- | --- | --- | --- |
|  |  | Area used (m^2^) | Number of nesting attempts | Nest density: N/10 m^2^ | Area used (m^2^) | Number of nesting attempts | Nest density: N/10 m^2^ |
| Pond 1–2 | 2014 | 175.85 | 11 | 0.626 | 133.36 | 9 | 0.68 |
|  | 2015 | 22.00 | 5 | 2.273 | 22.00 | 4 | 1.818 |
|  | 2016 | 86.00 | 4 | 0.465 | 36,02 | 3 | 0.833 |
|  | 2017 | 706.07 | 11 | 0.156 | 343.99 | 9 | 0.262 |
| Pond 3–4 | 2016 | 10.50 | 3 | 2.86 | NA | 2 | NA |
|  | 2017 | NA | 2 | NA | NA | 2 | NA |
| Pond 5 | 2014 | 984.06 | 13 | 0.132 | 984.06 | 13 | 0.132 |
|  | 2015 | 1002.37 | 22 | 0.219 | 708.59 | 17 | 0.240 |
|  | 2016 | 1133.46 | 25 | 0.221 | 901.08 | 21 | 0.233 |
|  | 2017 | 1571.62 | 27 | 0.172 | 1571.12 | 23 | 0.146 |
| Dam of ponds 5 and 6 | 2015 | NA | 1 | NA | NA | NA | NA |
|  | 2017 | 8.00 | 3 | 3.750 | NA | 2 | NA |
| Pond 7 | 2015 | 4.00 | 4 | 10.000 | 4.00 | 4 | 10.000 |
|  | 2016 | 74.00 | 3 | 0.405 | 74.00 | 3 | 0.405 |
| Dam of ponds 7 and 8 | 2015 | NA | 2 | NA | NA | 2 | NA |
|  | 2017 | NA | 1 | NA | NA | NA | NA |
| Pond 8 | 2015 | NA | 2 | NA | NA | 2 | NA |
|  | 2016 | NA | 2 | NA | NA | 2 | NA |
|  | 2017 | NA | 1 | NA | NA | 1 | NA |
| Pond 9 | 2016 | NA | 2 | NA | NA | 2 | NA |
|  | 2017 | 70.51 | 3 | 0.425 | 70.51 | 3 | 0.425 |
| Pond 10 | 2015 | NA | 1 | NA | NA | 1 | NA |
|  | 2016 | NA | 1 | NA | NA | 1 | NA |
|  | 2017 | 966 | 3 | 0.031 | 966 | 3 | 0.031 |
| Pond 11 | 2017 | 1642.04 | 3 | 0.018 | 1642.04 | 3 | 0.018 |

**Table S13.** Results of Tukey test of the comparison of the corrected inter-nest distances on the different nesting areas Table continues on next page

| Reference |  | t-value | p-value |
| --- | --- | --- | --- |
| Pond 1-2 | Pond 10 | -2.21 | 0.388 |
| Pond 1-2 | Pond 11 | -2.44 | 0.252 |
| Pond 1-2 | Pond 3-4 | -1.894 | 0.609 |
| Pond 1-2 | Dam 5-6 | -2.64 | 0.162 |
| Pond 1-2 | Pond 5 | -0.65 | 1 |
| Pond 1-2 | Dam 7-8 | -2.371 | 0.288 |
| Pond 1-2 | Pond 7 | -4.190 | <0.01 |
| Pond 1-2 | Pond 8 | -2.75 | 0.123 |
| Pond 1-2 | Pond 9 | -2.65 | 0.157 |
| Pond 10 | Pond 11 | -0.17 | 1 |
| Pond 10 | Pond 3-4 | 0.43 | 1 |
| Pond 10 | Dam 5-6 | -0.31 | 1 |
| Pond 10 | Pond 5 | 2.12 | 0.447 |
| Pond 10 | Dam 7-8 | -0.95 | 0.992 |
| Pond 10 | Pond 7 | -0.24 | 1 |
| Pond 10 | Pond 8 | -0.74 | 0.999 |
| Pond 10 | Pond 9 | -0.32 | 1 |
| Pond 11 | Pond 3-4 | 0.61 | 1 |
| Pond 11 | Dam 5-6 | -0.141 | 1 |
| Pond 11 | Pond 5 | 2.36 | 0.296 |
| Pond 11 | Dam 7-8 | -0.83 | 0.997 |
| Pond 11 | Pond 7 | -0.04 | 1 |
| Pond 11 | Pond 8 | -0.59 | 1 |
| Pond 11 | Pond 9 | -0.152 | 1 |
| Pond 3-4 | Dam 5-6 | -0.76 | 0.998 |
| Pond 3-4 | Pond 5 | 1.79 | 0.679 |
| Pond 3-4 | Dam 7-8 | -1.27 | 0.943 |
| Pond 3-4 | Pond 7 | -0.81 | 0.997 |
| Pond 3-4 | Pond 8 | -1.16 | 0.968 |
| Pond 3-4 | Pond 9 | -0.77 | 0.998 |
| Dam 5-6 | Pond 5 | 2.56 | 0.196 |
| Dam 5-6 | Dam 7-8 | -0.73 | 0.999 |
| Dam 5-6 | Pond 7 | 0.14 | 1 |
| Dam 5-6 | Pond 8 | -0.46 | 1 |
| Dam 5-6 | Pond 9 | -0.01 | 1 |
| Pond 5 | Dam 7-8 | -2.32 | 0.320 |
| Pond 5 | Pond 7 | -4.14 | <0.01 |
| Pond 5 | Pond 8 | -2.68 | 0.145 |
| Pond 5 | Pond 9 | -2.57 | 0.189 |
| Dam 7-8 | Pond 7 | 0.88 | 0.995 |
| Dam 7-8 | Pond 8 | 0.34 | 1 |

**Table S13** Continued

| Reference |  | t-value | p-value |
| --- | --- | --- | --- |
| Dam 7-8 | Pond 9 | 0.72 | 0.999 |
| Pond 7 | Pond 8 | -0.66 | 1 |
| Pond 7 | Pond 9 | -0.15 | 1 |
| Pond 8 | Pond 9 | 0.45 | 1 |

**Table S14.** The effects of years on the mean corrected inter-nest distances of successful nesting attempts at all nesting areas and at the two preferred nesting areas (pond 1–2 and pond 5) in the different years with Tukey-tests

|  | | Corrected inter-nest distances of successful nestings | | | |
| --- | --- | --- | --- | --- | --- |
|  | | All nesting areas | Two preferred nesting areas | | |
|  | | *t- value* | *p-value* | *t-value* | *p-value* |
| 2014–2015 | | 5.14 | <0.001 | 5.78 | <0.001 |
| 2014–2016 | | 1.58 | 0.389 | 1.84 | 0.253 |
| 2014–2017 | | 1.65 | 0.350 | 1.94 | 0.211 |
| 2015–2016 | | 4.31 | <0.001 | 4.74 | <0.001 |
| 2015–2017 | 4.58 | | <0.001 | 5.00 | <0.001 |
| 2016–2017 | 0.01 | | 1 | –0.01 | 1 |

**Table S15.** Yearly variation in the migration distances to the nest sites, and the number of all nesting attempts. In the year where there was only one nesting attempt, its migration distance from the shore of the pond is given as mean distance. In the year where there were two nesting attempts, their migration distance from the shore of the pond was given as the minimum and maximum value.

| Nesting area | Year | Number of all nesting attempts | Migration distance (m) from the pond to the locations of all nesting attempts | | |
| --- | --- | --- | --- | --- | --- |
|  |  |  | Mean | SD | Min. – max. |
| Pond 1–2 | 2014 | 11 | 6.20 | 3.20 | 1.16–10.24 |
|  | 2015 | 5 | 6.19 | 1.35 | 5.03–8.24 |
|  | 2016 | 4 | 8.68 | 5.31 | 5.34–16.58 |
|  | 2017 | 11 | 5.43 | 3.36 | 0.82–12.35 |
|  | 2014–2017 |  | 6.24 | 3.36 | 0.82–16.58 |
| Pond 3–4 | 2016 | 3 | 14.52 | 1.10 | 13.26–15.34 |
|  | 2017 | 2 | 18.84 | NA | 16.13–21.55 |
|  | 2014–2017 |  | 16.25 | 3.14 | 13.26–21.55 |
| Pond 5 | 2014 | 13 | 96.98 | 9.95 | 76.71–112.45 |
|  | 2015 | 22 | 96.26 | 11.34 | 81.18–115.68 |
|  | 2016 | 25 | 99.85 | 9.91 | 78.30–117.30 |
|  | 2017 | 27 | 100.56 | 12.34 | 78.95–122.38 |
|  | 2014–2017 |  | 98.73 | 11.05 | 76.71–122.38 |
| Dam of ponds 5 and 6 | 2015 | 1 | 16.98 | NA | NA |
|  | 2017 | 3 | 16.73 | 9.07 | 8.19–26.24 |
|  | 2014–2017 |  | 16.79 | 7.40 | 8.19–26.24 |
| Pond 7 | 2015 | 4 | 51.47 | 4.36 | 45.44–55.76 |
|  | 2016 | 3 | 32.62 | 7.07 | 27.27–40.64 |
|  | 2014–2017 |  | 43.39 | 11.30 | 27.27–55.76 |
| Dam of ponds 7 and 8 | 2015 | 2 | 3.60 | NA | 3.54–3.66 |
|  | 2017 | 1 | 4.26 | NA | NA |
|  | 2014–2017 |  | 3.82 | 0.38 | 3.54–4.26 |
| Pond 8 | 2015 | 2 | 41.15 | NA | 38.34–43.95 |
|  | 2016 | 2 | 45.07 | NA | 44.74–45.40 |
|  | 2017 | 1 | 42.81 | NA | NA |
|  | 2014–2017 |  | 43.05 | 2.80 | 38.34–45.40 |
| Pond 9 | 2016 | 2 | 54.91 | NA | 54.27–55.54 |
|  | 2017 | 3 | 48.23 | 6.19 | 41.09–52.26 |
|  | 2014–2017 |  | 50.90 | 5.72 | 41.09–55.54 |
| Pond 10 | 2015 | 1 | 16.17 | NA | NA |
|  | 2016 | 1 | 23.32 | NA | NA |
|  | 2017 | 3 | 39.77 | 10.34 | 29.90–50.52 |
|  | 2014–2017 |  | 31.76 | 13.42 | 16.17–50.52 |
| Pond 11 | 2017 | 3 | 62.83 | 39.21 | 34.53–107.59 |
|  | 2014–2017 |  | 62.83 | 39.21 | 34.53–107.59 |

**Tables S16.** Full model output of *lm* model of effect of the pine forest on the single clutching or returning females, differences between the distances of northern or southern pine forests, single clutching or returning females, and differences between years

|  | Estimate | Std. Error | t-value | p-value |
| --- | --- | --- | --- | --- |
| intercept | 5.6883 | 0.1561 | 36.43 | <0.001 |
| Forest (North) | -2.0889 | 0.1109 | -18.83 | < 0.001 |
| Nesting (Returning) | -0.2651 | 0.1157 | – 2.29 | 0.023 |
| Year (2015) | 0.0953 | 0.1828 | 0.52 | 0.603 |
| Year (2016) | -0.0302 | 0.1792 | -0.17 | 0.866 |
| Year (2017) | -0.1666 | 0.1750 | -0.95 | 0.343 |

**Table S17.** Comparison of returning females and single clutch females with inter-nest distances at the two preferred nesting areas and the distance from the northern pine forest. The values are in metre. SD: standard deviation, N: sample size.

|  | Returning females | | | Single clutch females | | |
| --- | --- | --- | --- | --- | --- | --- |
|  | mean | SD | N | mean | SD | N |
| Inter-nest distance at pond 1-2 | 18.39 | 14.96 | 3 | 41.49 | 47.33 | 98 |
| Inter-nest distance at pond 5 | 36.67 | 63.20 | 20 | 53.78 | 51.21 | 342 |
| Distance from northern pine forest | 11.53 | 4.05 | 34 | 13.08 | 4.82 | 53 |

**Table S18.** Effect of pine forest depending on the nest aggregation type (bunch/line), forest side, and year in a *lm* model.

|  | Estimate | Std. Error | t-value | p-value |
| --- | --- | --- | --- | --- |
| intercept | 5.5163 | 0.2351 | 23.46 | <0.001 |
| Forest (North) | -2.0174 | 0.1314 | -15.35 | <0.001 |
| Type (line) | -0.1389 | 0.1356 | – 1.03 | 0.309 |
| Year (2015) | -0.0200 | 0.2548 | -0.08 | 0.938 |
| Year (2016) | -0.0986 | 0.2475 | -0.40 | 0.692 |
| Year (2017) | -0.2028 | 0.2554 | -0.79 | 0.430 |

**Table S19.** Results of Tukey test of years on distances of the bunch and single line like nests from the edge of the pine forest north of the nesting area at pond 5 between the years.

| Differences between years | t-value | p-value |
| --- | --- | --- |
| 2014–2015 | 0.105 | 1 |
| 2014–2016 | 0.206 | 0.997 |
| 2014–2017 | 0.368 | 0.982 |
| 2015–2016 | –0.150 | 0.999 |
| 2015–2017 | –0.383 | 0.980 |
| 2016–2017 | –0.254 | 0.994 |

**Table S20.** Results of *lm* model of mean inter-nest distance differences between single clutching and returning females

|  | Estimate | Std. Error | t-value | p-value |
| --- | --- | --- | --- | --- |
| intercept | 6.3033 | 0.1579 | 39.93 | <0.001 |
| Return | -0.9444 | 0.6154 | – 1.54 | 0.126 |

**Table S21.** Results of *lm* model of mean inter-nest distance differences between single clutching and returning females. This case the returning female’s distances always calculated from the first nest.

|  | Estimate | Std. Error | t-value | p-value |
| --- | --- | --- | --- | --- |
| intercept | 6.2716 | 0.1558 | 40.25 | <0.001 |
| Return | -1.0095 | 0.7207 | – 1.40 | 0.162 |

**Table S22.** Differences between the inter-nest distances of single clutching females and returning females between the years. *Lm* model.

|  | Estimate | Std. Error | t-value | p-value |
| --- | --- | --- | --- | --- |
| intercept | 5.2621 | 0.6521 | 8.07 | <0.001 |
| 2014 | -1.1321 | 0.7389 | -1.53 | 0.126 |
| 2015 | -0.3255 | 0.7399 | -0.44 | 0.660 |
| 2016 | 1.2911 | 0.7333 | 1.76 | 0.079 |
| 2017 | 2.1668 | 0.6852 | 3.16 | 0.002 |

**Table S23.** Differences between the inter-nest distances of returning females compared to return in the same year (intercept) and return distances in the subsequent years. *Lm* model

|  | Estimate | Std. Error | t-value | p-value |
| --- | --- | --- | --- | --- |
| intercept | 28.993 | 10.464 | 2.77 | 0.010 |
| Return (1year) | 5.524 | 13.080 | 0.42 | 0.676 |
| Return (2year) | 17.049 | 17.509 | 0.97 | 0.339 |
| Return (3year) | -14.673 | 33.089 | – 0.44 | 0.661 |
